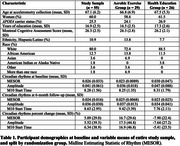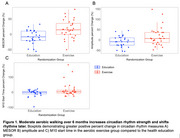# A 6‐month moderate aerobic walking program strengthens circadian rhythms in community‐dwelling older adults: The LEARNit trial

**DOI:** 10.1002/alz70856_099434

**Published:** 2025-12-24

**Authors:** Joanna L Eckhardt, A. Lisette Isenberg, Teresa Monreal, Joy Stradford, Laura E. Fenton, Joey A Contreras, Wendy J Mack, Judy Pa

**Affiliations:** ^1^ Alzheimer's Disease Cooperative Study (ADCS), University of California, San Diego, La Jolla, CA, USA; ^2^ Mark and Mary Stevens Neuroimaging and Informatics Institute, Keck School of Medicine, University of Southern California, Marina del Rey, CA, USA; ^3^ SDSU/UCSD Joint Doctoral Program in Clinical Psychology, University of California, San Diego, La Jolla, CA, USA; ^4^ University of Southern California, Los Angeles, CA, USA; ^5^ Keck School of Medicine, University of Southern California, Los Angeles, CA, USA

## Abstract

**Background:**

Exercise and circadian therapies are promising lifestyle interventions for reducing Alzheimer's disease risk and progression. This work investigates how 6‐months of aerobic exercise impacts circadian rhythms in older adults with objective early cognitive impairment.

**Method:**

This work utilized longitudinal data from the Lifestyle Enriching Activities for Research in Neuroscience Intervention Trial (LEARNit). The sample included 55 sedentary older adults ages 55‐79 with objective early cognitive impairment. Over 6 months, participants completed either 1) 150 minutes/week of moderate‐aerobic walking (*n* = 29) or 2) self‐paced reading of healthy lifestyles topics (*n* = 26).

Participants wore a GENEActiv (Activinsights Ltd) accelerometer for on average 31.8 ± 4.2 days (range: 21‐45 days) at baseline, and 25.3 ± 7.3 days (range: 10‐40 days) at 6‐month follow‐up. All analyses were conducted in R (version 4.3.1). Processing of raw accelerometer data was conducted with GGIR (version 3.0‐1). ActCR (version 0.3.0) was used to extract amplitude and Midline Estimating Statistic of Rhythm (MESOR), two measures of circadian strength. Amplitude refers to the activity oscillation amplitude, whereas MESOR is a rhythm‐adjusted mean of activity levels. nparACT (version 0.8) was used to extract M10 start time—the start time of the 10 hours with highest average amplitude. Percent change was quantified for these measures between baseline and 6‐months. Analysis of covariance (ANCOVA) were conducted to compare percent change between randomization groups, and were adjusted for age, sex, and *APOE4* carrier status.

**Result:**

Participant demographics are summarized and split by randomization group in Table 1. ANCOVA showed a significant difference in percent change of MESOR (*F*(1,50)=12.18, *p* = 0.0010 η^2^=0.18) and amplitude (*F*(1,50)=6.04, *p* = 0.018, η^2^=0.10) between groups, such that the exercise group increased both measures of circadian strength (Figure 1). There was also a significant difference in percent change of M10 start time (*F*(1,50)=4.64, *p* = 0.036, η^2^=0.083), such that the exercise group shifted M10 start time later (Figure 1).

**Conclusion:**

Moderate aerobic walking over 6 months resulted in a greater positive percent change in MESOR, amplitude, and M10 start time compared to the health education group. These results indicate that a moderate aerobic walking program, or exercise, in sedentary older adults can increase circadian strength and shift rhythm timing.